# Supplementary material for: MetaRibo-Seq measures translation in microbiomes
Source: Nat Commun. 2020 Jun 29;11:3268. doi: 10.1038/s41467-020-17081-z (PMC7324362; doi:10.1038/s41467-020-17081-z)
Supplement: Supplementary file 10 — Supplementary Data 7 [file 41467_2020_17081_MOESM10_ESM.zip › File2/Confidence_High_Taxonomy/202248_out.krona.html]

Javascript must be enabled to view this page.

members
magnitude
magnitudeUnassigned
count
unassigned
taxon
rank

202248\_out

45

superkingdom
45
2

45
976
phylum

45
200643
class

order
45
171549

815
3
family

genus
3
816


SRS893252\_contig\_number\_17395
2292944
1
species

1
371601

SRS064973\_contig\_number\_410
species

28111

SRS018575\_contig\_number\_7335
1
species

family
1853231
1

genus
283168
1

species

SRS058770\_contig\_number\_23363
28118
1

family
41
2005525

genus
10
375288
41

SRS014287\_contig\_number\_19882SRS019808\_contig\_number\_5575SRS045244\_contig\_number\_7394SRS048164\_contig\_number\_contig-100\_3150.136302SRS056519\_contig\_number\_15487SRS056537\_contig\_number\_1825SRS077294\_contig\_number\_contig-100\_2747.82571SRS143085\_contig\_number\_contig-100\_392.33493SRS144603\_contig\_number\_10604SRS148970\_contig\_number\_352

823

SRS014459\_contig\_number\_11340SRS015065\_contig\_number\_contig-100\_229.221655SRS015217\_contig\_number\_31586SRS015579\_contig\_number\_25437SRS015890\_contig\_number\_5820SRS018984\_contig\_number\_1771SRS019397\_contig\_number\_8493SRS019968\_contig\_number\_2557SRS046369\_contig\_number\_477SRS049995\_contig\_number\_29639SRS051610\_contig\_number\_10995SRS051882\_contig\_number\_10589SRS054590\_contig\_number\_230SRS062701\_contig\_number\_6088SRS075878\_contig\_number\_7230SRS077231\_contig\_number\_contig-100\_400.40191SRS077454\_contig\_number\_19357SRS1041091\_contig\_number\_16263SRS1041142\_contig\_number\_2491SRS1055038\_contig\_number\_6007SRS142712\_contig\_number\_2672SRS148511\_contig\_number\_16627SRS893382\_contig\_number\_6721
23
species

species
6

SRS015431\_contig\_number\_35473SRS019068\_contig\_number\_53025SRS020622\_contig\_number\_4381SRS051031\_contig\_number\_24250SRS063370\_contig\_number\_13010SRS1041090\_contig\_number\_5079
2293123

2
46503

SRS043667\_contig\_number\_1005SRS098717\_contig\_number\_7188
species
